# The Potential of Bacteriophage-Antibiotic Combination Therapy in Treating Infections with Multidrug-Resistant Bacteria

**DOI:** 10.3390/antibiotics12081329

**Published:** 2023-08-17

**Authors:** Abdul-Halim Osman, Fleischer C. N. Kotey, Alex Odoom, Samuel Darkwah, Raphael K. Yeboah, Nicholas T. K. D. Dayie, Eric S. Donkor

**Affiliations:** Department of Medical Microbiology, University of Ghana Medical School, Korle Bu, Accra P.O. Box KB 4236, Ghana; abdulhalimosman20@gmail.com (A.-H.O.); fleischarles@gmail.com (F.C.N.K.); alexodoom2018@gmail.com (A.O.); kwekuadarkwah@gmail.com (S.D.); yeboah.k.raphael@gmail.com (R.K.Y.); ntkddayie@ug.edu.gh (N.T.K.D.D.)

**Keywords:** antibiotic resistance, bacteria, bacteriophage, multidrug, phage, therapy, synergistic, probiotic, global health

## Abstract

The growing threat of antibiotic resistance is a significant global health challenge that has intensified in recent years. The burden of antibiotic resistance on public health is augmented due to its multifaceted nature, as well as the slow-paced and limited development of new antibiotics. The threat posed by resistance is now existential in phage therapy, which had long been touted as a promising replacement for antibiotics. Consequently, it is imperative to explore the potential of combination therapies involving antibiotics and phages as a feasible alternative for treating infections with multidrug-resistant bacteria. Although either bacteriophage or antibiotics can potentially treat bacterial infections, they are each fraught with resistance. Combination therapies, however, yielded positive outcomes in most cases; nonetheless, a few combinations did not show any benefit. Combination therapies comprising the synergistic activity of phages and antibiotics and combinations of phages with other treatments such as probiotics hold promise in the treatment of drug-resistant bacterial infections.

## 1. Introduction

Globally, the management of infectious diseases has been jeopardized by the emergence and dissemination of multidrug-resistant (MDR) bacteria [1,2,3]. This menace is more concentrated in western sub-Saharan Africa, as revealed in a recent comprehensive review on the global burden of antimicrobial resistance (AMR) [3]. Sadly, the discovery of newer antibiotics is slow-paced, with the limited effective ones teetering on the brink of obliteration [3,4]. Although there have been efforts and calls to hasten the development of newer antibiotics [5,6,7], the discoveries alone could hardly be deemed a panacea for subduing microbes in the “AMR war”. Moreover, the discovery processes, even if eventually successful, are capital-intensive and concurrently less financially rewarding in the contemporary scheme of pervasive microbial adaptations [8,9,10]. One revolutionary approach that has “made headlines” in the infectious diseases community is phage therapy [11,12,13]. However, the threat posed by resistance is now existential in this long-touted antimicrobial arsenal as well, making major solitary investments in this alternative blatantly imprudent [14,15,16,17]. In this review, we evaluate the prospects of harnessing the complementarity of phage and antibiotic therapies in mitigating the further foreseeable damage of AMR.

## 2. Rationale for Adopting Phage-Antibiotic Combination Therapy

The accidental discovery of penicillin by Sir Alexander Fleming in the early 20th century and its subsequent success in treating otherwise fatal infections marked the beginning of a golden age of effective resolution of infectious diseases [18,19,20]. This raised high expectations, which, however, needed to be immediately readjusted, as resistance to this drug surfaced not long after [21,22]. Following the major success of penicillin, additional antibiotics were mass-produced, but these met with an outcome similar to that of penicillin [23,24,25]. In the 21st century, the proportion of microbes resistant to hitherto effective drugs has become overbearingly high, and AMR has incidentally become a cliché, a sort of “new normal” [3,26,27]. Microbial resistance to one or two antibiotics now seems lower-tier, as the fractions of microbes classified as MDR, extensively drug-resistant, and pan-drug-resistant have been on a rapid rise [3,28]. The focal point of AMR discussions has, thus, shifted to the multidrug-resistance subcomponent of the AMR menace [1,26]. Moreover, in the recent comprehensive review on the global burden of AMR, 4.95 million deaths were associated with AMR, a fourth of which was directly attributed to the menace [3]. Worse yet, annual fatalities from AMR are expected to reach 10 million by 2050, with extreme economic upheavals occurring in more than double the number of fatal cases [29,30]. With good reason, the World Health Organization has classified AMR as a global public health threat, requiring urgent and sustainable remedial efforts [31,32].

One strategy that has been promoted as a plausible candidate for resolving the AMR menace is phage therapy. It involves therapeutic exploitation of the selective lethality of bacteriophages to bacteria—the phages infect a proportion of the infecting bacteria in vivo, and upon completion of each lytic cycle, yield a large collection of new phage virions to infect remnant bacteria. Several success stories have been reported in connection with this strategy in respiratory [33,34,35,36], wound [37,38,39], and even chronic [38,40] infections in its about a century-spanning usage [41,42]. It has also proven effective in clearing persister cells [43,44] and bacterial biofilms [45,46,47,48]. These successes notwithstanding, bacterial resistance to phages has also been reported, as occurred in the case of antibiotics [14,15,16,17]. This overlapping phenomenon highlights a feature of microbes that may not have been given much consideration at the time when the search for antimicrobials began—provocation-induced adaptations. Current therapeutic efforts targeted at microbes need to factor in their adaptive feature if rapid microbe-rendered obsoletion of these efforts is to be circumvented. In line with this, we “add our voice” to calls for the advances made in phage therapy to be combined with the relative efficacy of antibiotics to create a synergy that may be robust enough to foil, or mitigate at worst, survival induction in microbes [40,49,50,51,52,53,54,55].

## 3. Approaches to Phage-Antibiotic Combination Therapy

In phage-antibiotic combination therapy, or phage-antibiotic synergy (PAS), bacteriophages are used in combination with antibiotics, with phage-antibiotic interactions resulting in synergistic, antagonistic, or additive effects [51,56,57,58,59]. There are different approaches to combining antibiotics and phages, including using phages to enhance the activity of antibiotics and using antibiotics to prevent the emergence of phage-resistant bacteria. These approaches can be through sequential or concurrent/simultaneous administration of phage and antibiotics.

According to Chegini et al. [60], the combined use of phages and antibiotics with a sequential application demonstrated greater effectiveness in killing bacteria as opposed to simultaneous application. This finding was based on the observation that the highest inhibitory effect on biofilm was achieved when antibiotics were administered following phage treatment in a sequential manner. Biofilm destruction of *Pseudomonas aeruginosa* was moderately affected by using either antibiotics or phages exclusively. However, a significant improvement in killing efficacy was observed when phage vB_PaM_EPA1 and antibiotics were used together in a simultaneous or sequential manner [61]. Chaundry et al. [45] and others [62,63] provided evidence that the optimal way to hinder biofilm growth is by administering phages before antibiotics—the reason for this is the superior proliferation ability of phages in large bacterial populations, a scenario which is undermined when phages are introduced after antibiotic treatment. Hence, it appears that administering phages before antibiotic treatment is the recommended approach [60].

Another approach is to use phages to enhance the activity of antibiotics. This involves using phages to selectively target and kill antibiotic-resistant bacteria, thereby reducing the overall population of resistant cells and increasing the effectiveness of antibiotic treatment. For example, PAS is demonstrated to be an efficacious remedy for infections resulting from MDR *P. aeruginosa* [64]. Furthermore, phages can be used to deliver antibiotics directly to bacterial cells, elevating the antibiotic concentration at the infection site and reducing the risk of off-target effects [65].

In addition to the above, the use of antibiotics to prevent the emergence of phage-resistant bacteria is an approach worthy of mentioning. This approach involves using low-dose antibiotics to prevent the growth of bacterial cells that are resistant to phages, thereby reducing the risk of resistance development. For example, a combination of low-dose antibiotics and phages has been shown to be effective in preventing the emergence of phage-resistant bacteria in a model of a urinary tract infection [66].

One more approach to administering PAS involves using antibiotics to treat bacterial infections and phages to prevent reinfection or to reduce the risk of antibiotic resistance. This approach can help reduce the overall burden of antibiotic use and minimize the risk of resistance development. For example, PAS has been shown to be effective in reducing the risk of recurrent infections caused by antibiotic-resistant bacteria [67].

## 4. Phage-Antibiotic Combination Therapy: Success Stories, Challenges, Optimization, and Potential for Use in Resource-Limited Settings

### 4.1. Success Stories Involving Phage-Antibiotic Combination Therapy

Recent evidence has been accumulating to support the effect of PAS against bacterial infections, as summarized in Table 1 [57,58,59]. For instance, it has been demonstrated that antibiotics at doses lower than the minimum inhibitory concentration (MIC) can stimulate phage productivity, resulting in a decline in bacterial populations through PAS [66,67]. In addition to its synergistic effects, the combined approach may also restore antibiotic sensitivity, especially when the phage interacts with the bacterial drug efflux system. This is illustrated by phage OMKO1, which utilizes the outer membrane porin M (OprM) of multidrug efflux systems MexXY and MexAB as receptor-binding sites on MDR *P. aeruginosa* [68]. Besides these, a test on four unrelated phages podovirus KPP25, siphovirus KPP23, pbunavirus KPP22, and podovirus KPP21 (N4-like virus) in addition to 25 different antibiotics yielded promising results. It was found that except for KPP25, all the phages exhibited PAS with 5, 13, and 3 antibiotics, respectively [69,70].

In one study, Tkhilaishvili et al. [71] evaluated the use of PAS for the treatment of patients with MDR bacterial infections. The study found that the sequential combination of colistin 8 h after phage therapy was more effective than either treatment alone in reducing bacterial load and improving clinical outcomes. In another evaluation that involved treating methicillin-resistant *Staphylococcus aureus* (MRSA) infections [68], PAS was more effective in reducing bacterial load and preventing the emergence of phage-resistant bacteria than treatment involving the exclusive use of phages or antibiotics.

Importantly, PAS is known to be effective against biofilms, a hugely significant characteristic of bacterial resistance to many classes of antibiotics. For instance, a study by Pires et al. [72,73] demonstrated that the use of phages alone led to a notable decrease in the formation of biofilms by *P. aeruginosa*, while the combination of antibiotics and bacteriophages resulted in the complete eradication of the biofilm. Another study by Lu and Collins [74] found that the use of engineered bacteriophages combined with antibiotics was more effective in controlling biofilm-associated infections than either treatment alone. Similarly, Łusiak-Szelachowska et al. [53] showed that PAS was effective in reducing the biofilm mass of *Acinetobacter baumannii*, a pathogen that is often associated with healthcare-associated infections. In addition to the aforementioned lines of evidence, Rahman et al. [75], in a groundbreaking study, demonstrated that an appropriate combination of phages and antibiotics (in this case, rifampicin), can effectively reduce the amount of *Staphylococcus aureus* biofilm. This assertion was based on their observation that only 35% of bacteria survived after the synergistic use of rifampicin and phage SAP-26 (siphovirus, Phietavirus) on clinical isolates of *S. aureus* D43-a; treatment with phage alone and rifampicin alone resulted in the survival of 72% and 60% of the bacteria, respectively, indicating that the combination is more effective than single treatments. Furthermore, Kirby [76] reported in a study conducted on the clinical isolate *S. aureus* PS80 that the combination of gentamicin and phage SA5 (a Myoviridae Kayvirus) was more effective against the bacteria than either treatment alone after 72 h of treatment. The team attributed this synergistic effect to gentamicin inducing an aggregate phenotype in *S. aureus* cells, which while facilitating biofilm formation as a means of evading antibiotic activity, also rendered the cells more susceptible to phage attack. As a result, cell densities were ultimately reduced [76].

Jansen et al. [77] reported that the T4-like bacteriophage, KARL-1 represents a potential novel candidate for the treatment of MDR *A. baumannii* AB01infections, and its therapeutic efficacy may be enhanced by the addition of conventional antibiotics. They found that meropenem at a concentration of 128 and 256 mg/L significantly augmented the antibacterial activity of the phage (multiplicity of infection of 10^−1^). Contrastingly, ciprofloxacin was found not to support phage activity. Though some MDR *A. baumannii* confers resistance to the bactericidal activity of meropenem as it prevents cell wall biosynthesis, the co-presence of phage and meropenem may induce cellular stress responses that may enhance phage propagation resulting in shorter latent periods or larger burst sizes [77,78]. Thus, whiles there are positive results regarding PAS, not all the combinations are effective [79]. Hence further studies need to be conducted to ensure a specific selection of antibiotics and phages.

The feasibility of PAS has been demonstrated in successful preclinical and clinical settings as well. One example is a study by Comeau et al. [66], which evaluated the effectiveness of using PAS to treat mice that had been infected with *P. aeruginosa*. The results of the study showed that the combined treatment was more successful in decreasing the number of bacteria and enhancing the survival rates of the mice compared to using either treatment alone. Another example is the study by Lu and Collins [74], which focused on the treatment of *Escherichia coli* infections in vitro and in a mouse model. The study found PAS to be highly potent than either treatment alone in reducing bacterial load and preventing the emergence of phage-resistant bacteria.

In humans, successful therapy using PAS has been reported in the treatment of a patient infected with MDR *A. baumannii* in Poland and Russia [49,80]. Another study, based on an analysis of outcomes in 23 patients with deep periprosthetic joint infection (PJI) of the hip joint, supported the efficacy of PAS after obtaining a 95.5% rate of treatment response. This was observed following a simultaneous use of “*Staphylococcal* bacteriophage” at a dose of at least 10^5^ PFU/mL via puncture/injection to combat *Staphylococcus* spp. in the presence of cefazolin, vancomycin, and ciprofloxacin at doses of 2.0 g × 3 times/day for 2 weeks 1.0 g × 2 times/day for 4 weeks and 1.0 g × 2 times/day for 4 weeks, respectively [49]. Another piece of evidence is the successful treatment of a 67-year-old patient with a previous *P. aeruginosa* infection of the urinary tract. A customized Pyophage cocktail (Eliava, #051007) comprising six lytic phages, each at a titer of 10^6^ PFU/mL, was formulated to target *P. aeruginosa*. The patient’s bladder was treated with approximately 20 mL of the Pyophage cocktail every 12 h for a duration of 10 days. Meropenem (1 g twice daily) and colistin (polymixin E, 100 mg twice daily) were initiated on Day 6, with additional intravenous meropenem administered from Days 6 to 36 and colistin given from Days 6 to 10 [81,82]. This combination therapy was successful in clearing the patient’s infection, and there were no adverse effects reported. Recently, minocycline and a personalized phage cocktail against MDR *A. baumannii* were reported to be effective in a 68-year-old diabetic patient. The *A. baumannii* infection was eradicated, and clinical improvement was observed following the administration of multiple phage cocktails via intravenous route at different time points. The first cocktail consisted of AB-Navy97, AB-Navy71, AB-Navy4, and AB-Navy1, while the second cocktail contained AbTP3ϕ1 and AB-Navy71. In addition, a locally administered phage cocktail containing C2P24, AC4, C2P21, and C1P12, along with minocycline, eradicated the infection [67]. The combination therapy was successful in clearing the patient’s infection, and there were no adverse effects from the treatment. Moreover, Chan et al. [83] reported on a case study that described the therapeutic application of phage OMKO1 to treat a chronic *P. aeruginosa* infection in a patient with an aortic Dacron graft and an associated aorto-cutaneous fistula. After a single application of injectable 10 mL of phage OMKO1 (10^7^ PFU/mL) and ceftazidime (0.2 g/mL), the infection appeared to have resolved without any signs of recurrence 4 weeks post-procedure, suggesting that the PAS therapy was effective.

**Table 1:** A summary of reported phage treatment involving combination therapy.

Study/Case	Antibiotic (Dosages)	Bacteriophage (Dosages)	Treatment	Duration	Route of Administration	Single Phage/Phage Cocktail	Targeted Bacterium
Tkhilaishvili et al. [71]	Colistin (150 mg every 24 h)	Phage (10^8^ PFU/mL)	Sequentially (phage first, and then colistin after 8 h)	Two weeks	Local delivery system phage, but not specified, and intravenous treatment with colistin	Purified single phage	MDR *P. aeruginosa*
Lu et al. [74]	Ofloxacin (30 and 60 ng/mL), gentamicin, and ampicillin (5 μg/mL)	Engineered bacteriophages (lexA3) (10^8^ and 10^9^ PFU/mL)	Simultaneous	1–6 h	Not specified	Not specified	*E. coli* infections
Rahman et al. [75]	Rifampicin (0.6 mg/L)	Phage SAP-26 (10^8^ PFU/mL)	Simultaneous	2–24 h	Not specified	Phage SAP-26	*S. aureus* biofilms
Kirby et al. [76]	Gentamicin (100 × MIC)	Phage SA5 (10^7^ PFU/mL)	Not specified	24 h	Not specified	Phage SA5	*S. aureus*
Jansen et al. [77]	Meropenem (128 and 256 mg/L)	KARL-1 bacteriophage	Not specified	24 h	Not specified	Phage KARL-1	MDR *A. baumannii*
Fedorov et al. [49]	Cefazolin (2.0 g × 3 times/day for 2 weeks), and vancomycin (1.0 g × 2/day for four weeks) with daptomycin (0.5 g/day for 3 weeks)	Staphylococcal bacteriophage (At least 10^5^ PFU/mL)	Simultaneous	7–10 days	Puncture/injection	Staphylococcal bacteriophages (phage cocktails)	*Staphylococcus* spp.
Khawaldeh et al. [81,82]	Meropenem (1 g × 2/day), and colistin (100 mg × 2/day for 5 days)	Pyophage cocktail (Eliava, #051007) (10^6^ PFU/mL)	Sequential* (administration of phages and antibiotic and commencement of antibiotic on Day 6)	7 days	Bladder (local) and intravenous	Pyophage cocktail (#051007)	*P. aeruginosa*
Schooley et al. [67]	Minocycline	Multiple phage cocktails (C2P24, AC4, C2P21, and C1P12)	Sequential (phage first, and then antibiotic)	245 days	Intracavitary and intravenous	Multiple phage cocktails	MDR *A. baumannii*
Chan et al. [83]	Ceftazidime (0.2 g/mL every 8 h via intravenous route)	Phage OMKO1 (10^7^ PFU/mL)	Simultaneous *	5 days	Injectable	Phage OMKO1	*P. aeruginosa*

* Not specifically stated in the study, but deduced from the methodology.

### 4.2. Challenges with Phage-Antibiotic Combination Therapy

While PAS has shown promise in the treatment of bacterial infections, there are also several challenges and limitations that need to be addressed. A major one is the potential for interactions between antibiotics and phages. Antibiotics can interfere with phage replication and reduce the efficacy of phage therapy. Conversely, too, phages can potentially interfere with the activity of antibiotics [84]. Therefore, careful selection of antibiotics and phages, as well as optimization of the timing and dosing of the treatments, is necessary to maximize the efficacy of PAS. Another challenge is the potential for the development of resistance to both antibiotics and phages. While PAS can help prevent the emergence of phage-resistant bacteria, the use of antibiotics can also increase the risk of antibiotic resistance development [85]. Therefore, it is important to monitor for resistance development and to develop strategies for minimizing the risk of resistance. In this regard, the PAS can be based on mutually exclusive resistance mechanisms, such that when phages use efflux pumps as receptors, bacteria with reduced phage adsorption are more sensitive to antibiotics [68]. The use of PAS also has implications for reducing the emergence of resistant strains [86].

The use of PAS can be complicated by the need to isolate and identify effective phages for specific bacterial infections, as well as the potential for regulatory challenges in approving and administering phage therapy [67]. It is important to address the challenges and limitations associated with this approach in order to optimize its use in clinical settings.

In addition, the lack of computational methods that allow a proper description of the PAS phenomenon limits compassionate PAS use. In a previous study [87], the lysis score was used to describe the lysis activity of phages by observing changes in optical density over time. A range of specific lysis scores of 1–3 was assigned to the phages (the phage with the highest MOI was assigned 1, and the second highest scored 2) [87]. In this regard, the evaluation of phage activity is constrained to a narrow range but disqualified for phages with wider host ranges [88]. Similarly, methods implemented by Merabishvili et al. [89] and Cooper et al. [90] in their respective studies face enormous limitations [88]. However, recent studies have found the PhageScore methodology to be a promising strategy to assess the activity of PAS. This is evident as Grygorcewicz et al. [91] used *Acinetobacter*-infecting phages Aba-4, Aba-1, and vB_AbaP_AGC01, alongside meropenem, gentamicin, norfloxacin, ciprofloxacin, and fosfomycin to obtain a lysis curve of bacteriophages under antibiotic pressure. The result obtained using PhageScore showed that it can provide valuable insights into PAS. Similarly, the use of PhageScore method for the evaluation of phage lytic activity against bacterial host was found to be effective. This was observed after phages such as kayviruses (vB_SauM-D, vB_SauM-C, vB_SauM-A) and three T4-like phages (labelled as *T4-like A*, *B*, and *C*) were used against *S. aureus* and *E. coli* [88].

### 4.3. Optimizing the Use of Phage-Antibiotic Combination Therapy

PAS optimization involves a multifaceted approach that includes identifying effective phages, optimizing dosing and timing, and minimizing the risk of resistance development, as shown in Table 2. Ongoing research is focused on developing and refining these strategies to maximize the efficacy of phage therapy and combination therapy (phage-based therapy) in clinical settings. A strategy for optimizing the use of phage therapy is to identify and isolate effective phages for specific bacterial infections and optimize the dosing and timing of phage therapy. This can involve screening environmental samples for phages that target specific bacterial strains, as well as using phage libraries or synthetic biology approaches to engineer phages with specific properties [92]. Genetically engineered (GE) phages have the potential to be effective in treating bacterial infections, as they can be modified to encode antimicrobial peptides and toxins and can deliver their genetic load to infected cells to kill them or modify their metabolism [93]. They have a narrow host range, but this specificity can be redirected towards other bacteria by selecting GE phages that expose peptides that bind to them [94]. However, phage genetic engineering has limitations, and changes in the phage genome or virion structure can negatively affect infectivity or development. There are also concerns about the potential environmental and safety impacts of using GE phages in therapy [95]. Despite these concerns, studies have shown promising results in using GE phages to treat bacterial infections, and there have been successful cases of human phage therapy. One such successful case was reported by Dedrick et al.—a lytic derivative of a tested phage effectively eradicated a *Mycobacterium abscessus* infection in a cystic fibrosis patient [96]. Another study by Cobb et al. showed that a genetically modified *S. aureus* phage was effective in vitro in eliminating *S. aureus* and in vivo in reducing the bacterial burden in infected mouse skin and rat soft tissue infection models [97]. Feng et al. also demonstrated the efficacy of an engineered *S. aureus* phage JD419 in selectively killing only *S. aureus* strains carrying targeted virulence genes [98]. These studies provide evidence for the potential of engineered phages in treating bacterial infections.

Moreover, isolating spontaneous mutants on phage-resistant strains has also emerged as a promising approach to obtaining effective phages [99]. It relies on the inherent genetic variation of phages resulting from genome polymorphism within a population, which enables them to circumvent the anti-phage mechanisms of bacteria [99,100].

Currently, the concentration of phage for topical application ranges from 10^6^ to 10^9^ PFU/mL and for intravenous application, from 10^7^ to 10^11^ PFU/mL [101]. Therefore, determining the optimal dose requires more research to be able to obtain the optimal dose and frequency of many phage administrations, as well as identifying the most effective route of administration (e.g., intravenous, intraperitoneal, or topical) [67,101,102]. In the case of combination therapy, optimizing the timing and dosing of the treatments is also important to maximize their efficacy. This can involve selecting antibiotics that are compatible with phages and using them in a way that enhances the activity of phages, such as altering the physiological state of cells to filamenting cells and faster phage assembly [66]. Another important strategy for optimizing the PAS use is to minimize the risk of resistance development. This can involve using bacteriophages with a narrow host range to reduce the risk of phage-resistant bacteria emerging, as well as monitoring for resistance development and adjusting treatment strategies as necessary [103].

It is possible to increase the potential and activity of some phages. One team developed an injectable hydrogel that can encase *P. aeruginosa* bacteriophages (ΦW2005A, ΦPaer22, ΦPaer14, and ΦPaer4 each at 1.2 × 10^8^ PFU/mL and PsAer-9 at 3.0 × 10^4^ CFU) and transport them to the site of bone infections [104]. The bacteriophages retained their ability to destroy bacteria even after encapsulation and release from the hydrogel, and the rate of release could be regulated by adjusting the gel formulation. The bacteriophage-encapsulating hydrogels were effective in killing *P. aeruginosa* in both biofilm and planktonic forms in vitro, without impacting the metabolic function of human mesenchymal stromal cells. These hydrogels were also utilized to treat mice with *P. aeruginosa*-infected radial segmental defects, resulting in a 4.7-fold reduction in live *P. aeruginosa* counts at the infection site after 7 days, compared to hydrogels without bacteriophages. The findings suggest that bacteriophage-delivering hydrogels could be a promising optimized approach to treating localized bone infections [104]. Further, a potentially viable method for eradicating bacterial biofilms on surfaces has been demonstrated in the in vitro simultaneous application of bacteriophages (3 × 10^7^ PFU/mL) and chlorine disinfectants (210 mg/L) that increased the destruction of *P. aeruginosa* biofilm and facilitated the lysis of biofilm cells [105]. A study by Oliveira et al. [106] demonstrated the synergistic effect of honey and bacteriophages (vB_PaeP_PAO1-D and vB_EcoS_CEB_EC3a) in enhancing the antimicrobial activity of each other. The researchers observed that honey and phages could destroy bacterial biofilms through different mechanisms, which complemented each other. This was observed after studying ex vivo (porcine skin) and in vitro (polystyrene) models and against mono- and dual-species biofilms of *P. aeruginosa* and *E. coli*. The phages accomplished this by infecting and destroying bacteria through host-receptor recognition, while honey acts as an antimicrobial agent by inducing bacterial destruction through other mechanisms such as hydrogen peroxide release, acidity, osmotic pressure, oxidative stress, and the presence of methylglyoxal [106].

**Table 2:** Strategies for optimizing phage-antibiotic synergy therapy.

Strategies for Optimizing Phage-Antibiotic Combination Therapy	Examples	References
Step 1: Identify effective phages	Screen environmental samples for phages that target specific bacterial strains, or isolate spontaneous mutants on phage-resistant strains. Use phage libraries or synthetic biology approaches to engineer phages with specific properties.	[99,107,108]
Step 2: Optimize dosing and timing	Determine optimal dose and frequency of phage administration. Identify the most effective route of administration.	[107] [107,109]
Step 3: Select compatible antibiotics	Select antibiotics that are compatible with phages. Use antibiotics in a way that enhances the activity of phages, such as by weakening the bacterial cell wall.	[66,110]
Step 4: Minimize resistance development	Use bacteriophages with a narrow host range. Monitor for resistance development. Adjust treatment strategies as necessary.	[80,103]
Step 5: Increase phage activity	Develop injectable hydrogels that encase phages and transport them to infection site. Simultaneously apply bacteriophages and chlorine disinfectants. Use honey to enhance antimicrobial activity of phages.	[104,106]

### 4.4. Potential for Use of Phage-Antibiotic Combination Therapy in Resource-Limited Settings

PAS has a high potential to be used in settings with limited resources, where the development of new antibiotics may be difficult or expensive. In resource-constrained settings, the burden of antibiotic-resistant infections is often high, and access to effective antibiotics may be limited [111]. PAS provides an alternative strategy to address bacterial infections, particularly those caused by resistant strains. The phage therapy component is particularly attractive in resource-limited regions because it can be developed using relatively simple and inexpensive techniques. For example, bacteriophages can be isolated from the immediate surroundings and purified using basic laboratory equipment [67]. Furthermore, some bacteriophages can be preserved at room temperature and do not require expensive refrigeration or freezing equipment [67,112]. The merits of combination therapy involving phages and other antimicrobial agents, such as antimicrobial peptides or antibiotics may be best seen in resource-limited settings where AMR is more prevalent. For example, PAS is demonstrated to be effective in treating respiratory infections caused by antibiotic-resistant *P. aeruginosa* in animal models and others have been under clinical trials [113,114]. In addition, a combination of probiotics (*Enterococcus faecium*, *Lactobacillus casei*, *Lactobacillus acidophilus*, *Aspergillus oryzae*, and *Bifidobacterium termophilum*, each at 10^9^ CFU/g) and phage cocktail of *E. coli* P1, P2, P3, and P4 (about 10^10^ PFU/g) also demonstrated effectiveness in modulating gut microbiota of chicken to prevent and treat infections [115]. In an in vitro study, combination of Phage CA933P (5 × 10^8^ PFU/mL) and probiotic microbial mixture (made up of bacterial and yeast strains at concentrations of 10^9^ and 10^6^ CFU mL^−1^, respectively) in an attempt to treat Hep-2 cells-infected Enterohemorrhagic *E. coli* (EHEC) strains, such as EDL933 of EHEC O157:H7 (ATCC 700927), showed that the combination proved to be an effective treatment for EHEC infections, demonstrated by a reduction in the number of pathogens adhering to Hep-2 cells after 16 h [116].

There are challenges to the implementation of PAS in resource-limited areas. These include a lack of infrastructure and resources for research and development, limited access to diagnostic tools for identifying bacterial infections, and cultural barriers to the acceptance of phage therapy [117]. Despite these challenges, there is growing interest in the application of combination therapy. For example, several organizations, such as the Global Antibiotic Research and Development Partnership (GARDP), are working to develop and implement new phage therapies and combination therapies for the treatment of antibiotic-resistant infections in low- and middle-income countries (LMICs) [111].

## 5. Implications of Phage-Antibiotic Combination Therapy for Future Clinical Practice and Research

PAS has important implications for future clinical practice in the treatment of bacterial infections, as summarized in Table 3. One implication is the potential to provide alternative therapies for infections caused by bacteria that are resistant to antibiotics, as noted earlier. Clinical trials and case studies have provided evidence that these therapies are potentially effective in treating infections caused by MDR bacteria including *A. baumannii* and *P. aeruginosa* [67], biofilms, and mixed species biofilms [53,118]. For instance, Roszak et al. [119] reported a significant effect of PAS on biofilms composed of mono- or dual-species. The combined use of ciprofloxacin (1 mg/L) and phages caused a 90% and 69% reduction of biofilm-specific activities (BSA) of mono- and dual-species biofilms, respectively, while the single use of phages and that of ciprofloxacin recorded a lower reduction of BSA. This was observed after 24 h following the use of vB_SauM-A and vB_SauM-D phages (10^7^ PFU/mL) with ciprofloxacin (most effective conc. 16 to 32 mg/L) against *S. aureus* and *C. albicans.* Similarly, high PAS activity with limited persister cell regrowth was reported after 6 hours of combined treatments by Grygorcewicz et al. [120] when phage cocktail of (Aba-6, Aba-4, Aba-1, Aba-2, and Aba-3) at a final concentration of 10^9^ PFU/mL in combination with trimethoprim/sulfamethoxazole, was used against 25 strains of MDR *A. baumannii* biofilms. Positive effects were also observed for tobramycin, imipenem, and meropenem [120]. In another study, antibiotics (meropenem and ciprofloxacin) in combination with phage vB_AbaP_AGC01 resulted in PAS that improved the therapeutic efficacy of phage therapy against *A. baumannii* in vivo models (*G. mellonella* larvae and human heat-inactivated plasma blood model) by increasing the larval survival from 35% to 77% [121].

Another implication is the need for standardized protocols and guidelines for the safe and effective use of bacteriophage therapy in clinical settings. The development of these guidelines, as well as the establishment of regulatory frameworks for the approval and use of phage-based products, will be important for ensuring the safety and efficacy of these therapies [103]. In addition, the development of phage-based therapy has the potential to shift the focus of treatment from broad-spectrum antibiotics to targeted therapies that are specific to the bacterial strain causing the infection. This could help reduce the risk of resistance development and improve the overall effectiveness of treatment [85]. Ongoing research and collaboration among researchers, clinicians, and regulatory agencies will be important for further refining these therapies and optimizing their use in clinical settings. Future directions for research in phage-based therapy should include developing new strategies for identifying effective phages, optimizing dosing and administration protocols, and investigating the use of combination therapy in different clinical settings.

One area of research is focused on developing new strategies for identifying effective phages. This includes the use of high-throughput screening methods to identify phages with specific properties, as well as the development of synthetic biology approaches to engineer phages with enhanced properties [74,92]. A compelling piece of evidence of this is the treatment of a patient with a MDR *M. abscessus* infection. The individual was intravenously treated with a genome-engineered phage, and the infection was effectively cleared with no adverse effects [96]. Another area is focused on optimizing dosing and administration protocols for phage-based therapy. This includes investigating the optimal dose and frequency of phage administration and identifying the most effective route of administration for different infections [67,96]. Additionally, there is a need for further research into the use of combination therapy in different clinical settings, such as in the treatment of chronic infections or infections caused by MDR bacteria. This includes investigating the efficacy and safety of different combination therapy regimens, as well as developing strategies for minimizing the risk of resistance development [85].

Ongoing research in phage-based therapy is focused on developing new strategies for identifying effective phages, dose optimization, and administration protocols, and expanding the use of these therapies in different clinical settings. Further research is needed to fully realize the potential of phage and combination therapies in the treatment of bacterial infections.

## 6. Conclusions and Future Perspectives

The rapid rise and dissemination of bacterial multidrug resistance have rendered the exclusive use of antibiotics in infectious disease treatment inevitably near-obsolete. Investing research efforts in solitary therapeutic replacements, such as phage therapy, could not be considered a “safe haven”. However, as phage therapy has shown great promise during its century-spanning applications, its positives could be leveraged along with those of antibiotics to yield a robust synergy of combination therapy. Admittedly, phage-antibiotic combination therapy is undoubtedly not fool-proof, but it can potentially be modeled to be near-perfect. Such a highly efficient set of biosystems could be further exploited in augmenting antiviral, antifungal, and antiparasitic therapy. It is only after attaining such a feat would mankind be close to winning the seemingly insurmountable “AMR war”.

## Figures and Tables

**Table 3:** Ongoing research in phage-based therapy.

Focus of Research	Description	References
Identify effective phages	Developing new strategies to identify phages that are effective against a broader range of bacterial strains and species, including those that are resistant to antibiotics.	[92,122,123]
Optimize dosing and administration protocols	Investigating the optimal dosing and administration protocols for phage therapy and combination therapy, including the use of different routes of administration, treatment durations, and dosages.	[67,96,124,125]
Expanding use in different clinical settings	Exploring the potential use of phage therapy and combination therapy in different clinical settings, including hospitals, long-term care facilities, and outpatient clinics, and identifying the most appropriate patient populations for these therapies.	[67,126,127]
Developing new phage and combination therapies	Developing new phages and combination therapies that are effective against a broader range of bacterial strains and species, including those that are MDR, and optimizing the production and quality control processes for these therapies.	[83,110,122]
Understanding mechanisms of action	Investigating the mechanisms of action of phages and combination therapies and how they interact with the bacterial host, the immune system, and other factors that may influence treatment outcomes.	[66,128,129]
Clinical trials	Conducting clinical trials to evaluate the safety and efficacy of phage therapy and combination therapy in different patient populations and settings and comparing their outcomes to those of standard antibiotic therapy.	[67,130,131]

## Data Availability

Not applicable.
